# Irregular Migration as a Potential Source of Malaria Reintroduction in Sri Lanka and Use of Malaria Rapid Diagnostic Tests at Point-of-Entry Screening

**DOI:** 10.1155/2013/465906

**Published:** 2013-06-19

**Authors:** Kolitha Wickramage, Gawrie N. L. Galappaththy, D. Dayarathne, Sharika L. Peiris, Rajeeka N. Basnayake, Davide Mosca, Jan Jacobs

**Affiliations:** ^1^Health Unit, International Organization for Migration (IOM), No. 62, Green Path, Ananda Coomaraswamy Road, 3 Colombo, Sri Lanka; ^2^Anti-Malaria Campaign, Ministry of Health & Global Fund for AIDS, TB and Malaria, No. 555/5, Public Health Building, Narahenpita, 5 Colombo, Sri Lanka; ^3^Batticaloa Teaching Hospital, Hospital Lane, 30000 Batticaloa, Sri Lanka; ^4^Migration Health Department, International Organization for Migration (IOM), 17 Route des Morillons, 1211 Geneva 19, Switzerland; ^5^Department of Clinical Sciences, Institute of Tropical Medicine (ITM), Nationalestraat 155, 2000 Antwerpen, Belgium

## Abstract

*Background*. We describe an irregular migrant who returned to Sri Lanka after a failed people smuggling operation from West Africa. *Results*. On-arrival screening by Anti-Malaria Campaign (AMC) officers using a rapid diagnostic test (RDT) (CareStart Malaria HRP2/PLDH) indicated a negative result. On day 3 after arrival, he presented with fever and chills but was managed as dengue (which is hyperendemic in Sri Lanka). Only on day 7, diagnosis of *Plasmodium falciparum* malaria was made by microcopy and CareStart RDT. The initially negative RDT was ascribed to a low parasite density. Irregular migration may be an unrecognized source of malaria reintroduction. Despite some limitations in detection, RDTs form an important point-of-entry assessment. As a consequence of this case, the AMC is now focused on repeat testing and close monitoring of all irregular migrants from malaria-endemic zones. *Conclusion*. The present case study highlights the effective collaboration and coordination between inter-governmental agencies such as IOM and the Ministry of Health towards the goals of malaria elimination in Sri Lanka.

## 1. Introduction

Malaria is an important disease along international and internal borders that continues to contribute to a large burden of disease in the South East Asian Region (SEAR). Due to extensive efforts, progress is being made. During the 2000–2011 period, the number of confirmed cases of malaria declined by 24% and deaths by 68%. Of the 10 malaria-endemic countries in the region, Sri Lanka has reached elimination phase of malaria, whereas Bhutan is in pre-elimination phase.

Sri Lanka has been heralded as a success story in malaria control in Asia [1]. In 2008, Sri Lanka entered the preelimination phase of malaria control [2]. The slide positivity rate declined from 2% in 1999 to less than 0.1% in 2011, indicating a significant reduction in transmission. 

Infection is mostly encountered among travelers who return from endemic countries or among military personnel serving in the northeast of the country [3, 4]. The present case study highlights a newly recognized route of entry for malaria to Sri Lanka through returning “irregular migrant” flows. In global context, the term “irregular migration” typically refers someone who, owing to illegal entry or the expiry of his or her legal basis for entering and residing, lacks legal status in a transit or host country. The term applies to migrants who infringe a country's admission rules and any other person not authorized to remain in the host country.

These routes may act as potential source of malaria reintroduction, retarding elimination goals of the nation's Anti-Malaria Campaign (AMC). Rapid diagnostic tests (RDTs) for malaria if performed correctly offer excellent diagnostic capability for screening of “at-risk” groups at ports of entry [5]. However, as this case study illustrates, they have several limitations. 

## 2. Case Report

*Background and Travel History*. A 42-year-old male farmer of Tamil ethnicity from the Eastern Province of Sri Lanka joined a group of other irregular migrants that left on a flight to Benin in December 2011. All had paid large sums of money to a human smuggler, who had assured them safe passage to Canada and legal work permits on arrival. They arrived in Mali on 25th of December 2011 where another group of people smugglers arranged them to stay in a small shelter for one month. After a month, they travelled via flight to Benin, where they joined other cohorts of irregular migrants from Sri Lanka. The shelters they lived in had only basic facilities with no protection from mosquitoes. The smugglers intended to reach a quota of at least 900 people from Sri Lanka before charting a fishing vessel to enter Canadian ports. The scheme had already proven successful on previous occasions. During his time in Benin, the farmer recalled a heavy presence of mosquitoes and regular episodes of fever among the smuggled cohort. Smugglers had refused medical assistance in fear of alerting authorities. One death of a person due to “fever-like illness” was also reported although no medical details were available. The captives managed to escape and had alerted domestic and international law enforcement authorities who eventually informed the International Organization for Migration (IOM) to support the safe return of all irregular migrants from West Africa to Sri Lanka. 

Upon arrival at Bandaranaike International Airport in Colombo (day 1), all irregular migrants escorted by IOM were screened by Anti-Malaria Campaign officers (AMC) of the Ministry of Health. According to AMC standard procedure, screening for Malaria was done by a rapid diagnostic test (RDT) and CareStart Malaria HRP2/PLDH (AccessBio Inc., Monmouth, USA, further referred to as CareStart) for the rapid qualitative determination of *Plasmodium falciparum*-specific histidine-rich protein 2 (HRP2) and pan-*Plasmodium*-specific parasite lactate dehydrogenase (pLDH) performed on venous blood. For those with a positive RDT, the procedure prescribed further microscopic examination of blood smears at the National Malaria Reference Laboratory; patients with *P. falciparum* are treated with artemether-lumefantrine (Coartem) for 3 days and primaquine stat dose. For *Plasmodium vivax *infections, treatment consists of chloroquine for 3 days and primaquine for 14 days. All infected patients are treated at a specialist Infectious Disease Hospital in Colombo, and in case of children less than 12 years, at the Lady Ridgway Children's Hospital. Since the current patient showed a negative result for the CareStart RDT, he was allowed to proceed home.

**Figure 1:**
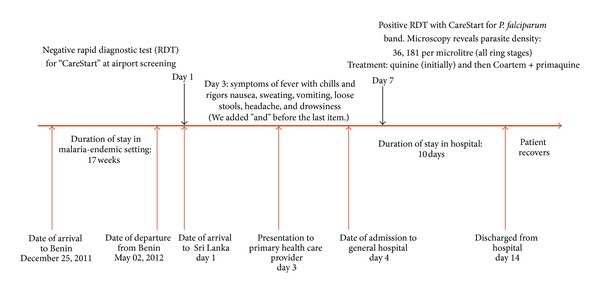
A timeline indicating key events of the patient.

On day 3 after arrival, the patient presented to a private clinic with symptoms of high fever, headache, chills, rigors, and preceding cold sweats. He was referred on day 4 to a Government Peripheral Hospital with suspicion for dengue fever. In view of clinical suspicion for rapid dengue progression, the attending doctor transferred the patient to Batticaloa Teaching Hospital in line with current policy of dengue management in Sri Lanka [6]. A timeline indicating key events of the patient is presented in Figure 1.

*Hospital Course*. For 48 hours upon admission, the patient received supportive treatment for the presumed diagnosis of dengue infection. As part of treatment follow-up, platelet counts were daily measured. Due to low platelet counts during the first two to three days, the initial suspicion was maintained for dengue. However, thrombocytopenia due to *Plasmodium* infection was suggested after the dengue antibody test showed a negative result and when the platelet count began to rise again after two days of hospital admission. By the end of day 7, venous blood was submitted to the hospital laboratory for RDT testing (CareStart) and microscopic examination. The slides were later sent to reference laboratory at AMC in Colombo for confirmation. Giemsa-stained thick and thin blood films were examined for the detection of malarial parasites. The RDT was positive for *P. falciparum* and was confirmed by the microscopy (double chromatin ring stage and “appliqué” form of trophozoites), and the parasite density was calculated as 36,180 asexual parasites/*µ*L, which equals 0.7% of red blood cell infected. CareStart RDT revealed the presence of a visible HRP2 and pan-pLDH test line.

Once the diagnosis of *P. falciparum* was made, intravenous quinine (800 mg q8h) was administered. However, this protocol was changed to treatment according to AMC guidelines, that is, artemether-lumefantrine (Coartem) for 3 days and primaquine stat dose. Daily followup was carried out for the early detection of complications and the therapeutic response to drugs. Parasites disappeared within 5 days of treatment, and the patient recovered well and was discharged at day 14. There was no recrudescence at the followup 10 days after the start of treatment. 

## 3. Discussion

The present report highlights the delay of malaria diagnosis in an immigrant returning from a malaria-endemic region to a country in the near-elimination phase of malaria.

*RDTs Do Not Completely Rule Out Malaria*. Despite microscopy remaining the “gold standard” for malaria diagnosis, studies in nonendemic and endemic countries have proven the effectiveness of RDTs [7]. A recent Cochrane systematic review concluded that RDTs “can replace or extend the access” of diagnostic services for uncomplicated malaria [8]. As some countries succeeded in reducing malaria prevalence and are moving towards malaria elimination, detection of low parasite densities has become increasingly important for diagnosis and clinical management [9]. For this reason, RDT test performance at low parasite densities is particularly important. A laboratory evaluation of CareStart on stored clinical samples showed detection rates of 90% for both *P. falciparum* and *P. vivax*. at parasite densities of 200 asexual parasites/*µ*L [10]. Another study in a reference setting showed diagnostic sensitivities for *P. falciparum* at parasite densities above 100 and 1,000/*µ*L to be 94.3% and 99.3%, respectively; for the detection of *P. vivax*, overall sensitivity was 77.6%, increasing to 90.2% at parasite densities above 500/*µ*L [11].

Malaria RDTs are a cost-effective and convenient screening technology, particularly at use in “point-of-entry” settings such as international airports. However, RDTs do have several limitations as described in this present case. We hypothesize the most likely cause for the initially negative CareStart RDT in the patient upon arrival at Colombo airport to be low parasite density. Indeed, the diagnostic sensitivity of CareStart RDT for the detection of *P. falciparum* dips to 69.9% at parasite densities below 100/*µ*L [11]. At the time of testing at airport, the levels may well have been well below 100 parasites/*µ*L.

As a consequence of this case, the AMC revised its follow-up procedure by undertaking repeat RDT testing of all irregular migrants channeled via IOM within 2 weeks of their arrival at home. This practice has since been extended to Sri Lanka's United Nations Peace Keepers returning from endemic areas. A network of AMC program officers and IOM staff at district level also conduct followup of the returnee cases.

*Malaria Should Not Be Overlooked in Patient Management*. Sri Lanka is also a hyperendemic country for dengue with repeated outbreaks occurring throughout the year [12]. Due to the rarity of malaria cases over the past decade, patients presenting with acute febrile illness and thrombocytopenia may lead clinicians to the diagnosis of dengue fever, or less commonly, leptospirosis [3]. However, the present patient history illustrates that awareness of malaria should be maintained in order to keep the diagnostic delay as short as possible. Clinical and laboratory competence can be improved and awareness can be triggered by various means such as prompt notification, continuous medical education, and external quality assessments.

*Implications for Malaria Elimination*. An increasing trend of irregular migrant flows has been reported in Sri Lanka since the end of civil conflict in 2009. IOM estimates a total of 900 Sri Lankans to be stranded in West Africa in 2012 alone. From January to July 2012, 14 cases of *Plasmodium falciparum* were detected in 437 returnees from people smuggling operations from West Africa [13]. Beyond the criminal and human rights implications for the victims of human smuggling/trafficking, there are serious public health concerns of malaria importation and reemergence. Surveillance of inbound migrant flows from endemic areas is vital to prevent reintroduction and re-emergence of malaria in Sri Lanka, especially since the country enters the malaria elimination phase. There has been limited attention to this route of importation by health authorities. 

As a consequence of this case, AMC revised its follow-up procedure by undertaking repeat RDT testing of all irregular migrants channeled via IOM within 2 weeks of their arrival at home. This practice has since been extended to Sri Lanka's United Nations Peace Keepers returning from endemic areas. The case study also highlights the importance of effective collaboration and coordination between intergovernmental agencies such as IOM and Ministry of Health towards the goals of malaria elimination by the end of 2012 [2].
